# Caregivers' perspectives on the in-home implementation and effectiveness of “Miffy eats the rainbow!”: a colorful, modeling- and reward-based intervention to improve fruit and vegetable intake in children

**DOI:** 10.3389/fpubh.2025.1663525

**Published:** 2026-01-12

**Authors:** Zoë S. van der Heijden, Gertrude G. Zeinstra, Femke J. de Gooijer, Elske M. Brouwer-Brolsma, Guido Camps

**Affiliations:** 1 Division of Human Nutrition & Health, Wageningen University & Research, Wageningen, Netherlands; 2 Wageningen Food & Biobased Research, Food, Health & Consumer Research Group, Wageningen University & Research, Wageningen, Netherlands; 3 OnePlanet Research Center, Wageningen, Netherlands

**Keywords:** children, fruit, vegetables, intervention, home-based, implementation

## Abstract

**Background:**

Home-based interventions can support fruit and vegetable (FV) consumption in young children. Although their effectiveness is well documented, real-life implementation experiences remain understudied; such knowledge is crucial for sustained and scalable impact. Citizen science offers a promising approach for collecting large-scale, practice-based insights into how such interventions are used in daily life.

**Objective:**

This study used a contributory citizen science approach to explore caregivers' experiences with the in-home implementation of “Miffy eats the rainbow!” and their perceptions of its effectiveness in supporting children's FV acceptance at home.

**Methods:**

A total of 42,000 sticker sheets were distributed across 420 Dutch retail locations, supported by a national media campaign. Caregivers who used the method at least once were invited to complete an online questionnaire assessing implementation outcomes (acceptability, appropriateness, feasibility, sustainability) and perceived effects on children's FV enjoyment, variety, and amount. Quantitative data were analyzed using descriptive statistics and ANOVA (by food fussiness); qualitative responses were thematically analyzed using an inductive approach.

**Results:**

A total of 209 caregivers participated (response rate: 0.5%). Implementation experiences were rated positively, with over 90% agreeing the method was fun (4.5 ± 0.6), easy to use (4.4 ± 0.7), and time efficient (4.3 ± 0.7). Ratings remained consistently high across all food fussiness levels. Perceived effects on children's FV enjoyment, amount, and variety were slightly positive, with mean scores ranging from 3.2 ± 1.0 to 3.6 ± 1.0. Qualitative responses provided additional insights into benefits, implementation strategies, and barriers.

**Conclusions:**

“Miffy eats the rainbow!” was positively received by self-selected caregivers, with high implementation ratings suggesting potential for broader in-home application. However, the very low response rate highlights challenges in achieving voluntary participation and raises concerns about selection bias. Future efforts should aim to promote inclusive engagement and evaluate long-term use across more diverse populations.

## 1 Introduction

Promoting fruit and vegetable (FV) intake in children is essential for healthy growth, cognitive development, and reducing the risk of later-life non-communicable diseases (1–3). Despite clear dietary guidelines and the well-established health benefits of FVs, intake among children remains insufficient (4). In the Netherlands, only 40% of children aged 4–9 meet the recommended intakes for fruits (≥150 g) and vegetables (≥100–150 g), with consumption declining as children age (5). Similar trends are observed in other Western countries (4, 6). Since dietary preferences and behaviors in childhood often persist into adulthood and influence long-term health outcomes (7, 8), there is a pressing need for scalable, easy-to-implement, and evidence-based interventions to improve FV intake.

Several school-based or family-based initiatives to stimulate FV intake have been studied (9–11), varying in both theoretical foundations and effectiveness. Among the strategies used, repeated exposure (12–14), modeling (13, 15–17), and non-food rewards (13, 15–18), have consistently shown positive effects on children's FV consumption, with evidence suggesting that combining these strategies enhances impact (11, 13). “Miffy eats the rainbow!” is a simple, colorful method that integrates these principles to promote FV tasting in young children (19). The method was previously evaluated in school settings, where structured eating moments provide a practical context for the intervention (20, 21). A nested cluster-randomized multi-arm trial demonstrated that the method significantly increased children's willingness to taste FVs compared to a control group (19). However, as 70% of children's daily caloric intake occurs at home (22), the home environment remains a crucial yet understudied setting for influencing dietary behaviors (23), but further research is needed to evaluate the method's feasibility and effectiveness in this context.

For FV interventions to achieve their intended impact, effectiveness alone is not sufficient; successful implementation is essential to translate interventions into sustained behaviors. Research has shown that implementation quality directly affects intervention outcomes and thereby their success in real-world settings (24). Gaining insight into whether a lack of effect results from poor design or inadequate delivery is key to accurate interpretation of findings from studies and to identify factors that facilitate or hinder effective use (24–26). However, most studies on FV interventions continue to emphasize outcomes such as increased liking, variety, or intake (10, 11, 13, 14, 18), while evaluations of implementation under real-life conditions remain limited (24, 26–28). Addressing this gap is vital to ensure that interventions proven effective can also be applied, sustained, and scaled in practice.

One way to advance implementation research in real-world settings is through using citizen science approaches, where researchers actively engage end-users (often referred to as “citizen scientists”) in scientific research (29). Citizen science, as part of the broader open science movement, emphasizes collaboration with or by the public rather than on the public (30). In health promotion, citizen science has gained traction over the past decades by expanding research reach, integrating community perspectives, and increasing public awareness and acceptance of interventions, including in the field of nutrition (31–33). Importantly, citizen science represents a spectrum of public involvement, ranging from contributory (e.g., citizen scientists primarily collect data), collaborative (e.g., citizen scientists are involved across multiple research stages), and citizen-led or co-created models (e.g., citizen scientists share control over the research process) (31, 34, 35). These approaches offer valuable opportunities to gather large-scale, practice-based data directly from caregivers, providing real-world insights into the implementation of interventions like “Miffy eats the rainbow!”; information often difficult to capture through traditional research designs.

Building on the need for real-world implementation research, this study used a contributory citizen science approach to examine how caregivers experience the use of “Miffy eats the rainbow!” in their home context. It investigates their perspectives on the intervention's acceptability, appropriateness, feasibility, and sustainability, as well as its perceived effectiveness in increasing FV consumption pleasure, variety, and intake. By engaging end-users in the evaluation process, this study contributes to a better understanding of how FV interventions can be effectively implemented and maintained in real-world family contexts.

## 2 Materials and methods

This study used a web-based, post-test only survey within a contributory citizen science framework to assess caregivers' implementation experiences and perceived effectiveness of the “Miffy eats the rainbow!” method for young Dutch children (31, 35).

### 2.1 Intervention materials: “Miffy eats the rainbow!”

“Miffy eats the rainbow!” is a simple, colorful method designed to encourage FV consumption in young children through modeling, repeated exposure, and non-food rewards, featuring Miffy (“nijntje” in Dutch). The materials and instructions for use have been described in detail in a previous study (19). In brief, caregivers read a short Miffy-themed story with their child, after which the child was encouraged to try or consume an FV from one of five color categories (red, orange, yellow, green, and blue). Attempts were rewarded with a corresponding sticker, which the child could place on a rainbow-shaped Miffy poster to reinforce progress and repetition.

### 2.2 Study procedures

A total of 42,000 sticker sheets were distributed across 420 branches of Bruna (www.bruna.nl) and De ReadShop (www.readshop.nl), two well-known book and office supply store chains in the Netherlands. Distribution was facilitated through the existing dissemination infrastructure of Mercis bv to ensure nationwide reach and availability across all provinces. Each store received 100 sticker sheets, which could be collected by caregivers free of charge from March 11–24, 2024 (1 per child). To improve accessibility, caregivers who were unable to collect a sticker sheet in-store due to physical or practical constraints could request one via email from the coordinating researcher, with 913 extra sticker sheets distributed this way. Caregivers who requested a sticker sheet by email also received a reminder to participate after 4 weeks. Each sticker sheet included a QR code on the back, directing caregivers to a dedicated webpage on the Wageningen University & Research (WUR) website, where they could download and print the poster and access the questionnaire. During the 2-week distribution period (March 11–24, 2024), the study webpage was visited 11,236 times by 7,059 unique users. To gain a general indication of actual distribution to caregivers, 10 participating stores from 7 out of 12 provinces were contacted approximately 2 weeks after the distribution period, based on suggestions from the distribution partner. Four stores (40%) reported that all sticker sheets were collected; the remaining stores (60%) reported distribution of at least 50%. Exact numbers across all stores were not available.

Caregivers were instructed to use the method at least once with their child(ren) before completing the questionnaire. They were given autonomy in how they implemented the method, including which eating moments to use it for, over what period, whether to apply it to both FVs or only one category, whether to include familiar or unfamiliar FVs, and whether to use for all colors or a selection of colors. However, to support those who wanted additional guidance, an instruction document was provided on the website, detailing: (1) required materials for using the method; (2) usage instructions (i.e., reading the story, rewarding the child with a sticker matching the color of the tried or consumed fruit or vegetable, and placing the sticker on the poster to complete the rainbow), including examples of FVs for each color; (3) a request to complete the questionnaire; and (4) practical tips for using the method. As an incentive to promote participation and completing the questionnaire, respondents who completed the questionnaire had a chance to win a visit to the Miffy Museum in Utrecht, including an overnight stay in a Miffy-themed hotel room for four people. Additionally, for every 100 respondents, a Miffy book package was raffled.

### 2.3 Questionnaire

Data was collected from March 2024 to November 2024 using a study-specific questionnaire designed to align with the research objectives. To minimize user drop-off and reduce unnecessary clicks, the questionnaire was hosted directly on the WUR website, avoiding redirection to external platforms (36). The questionnaire used conditional logic, with participants receiving a maximum of 50 questions depending on their responses; 32 were mandatory. Completion time was approximately 10 min for those receiving the full set. To minimize order bias, question order was randomized over time. Usability and comprehensibility were evaluated through pilot testing with end-users (*n* = 31, results not published) and think aloud interviews (*n* = 9, results not published) (37), supporting face validity (38). The complete questionnaire can be found in Supplementary File 1.

#### 2.3.1 Outcome measures

##### 2.3.1.1 Implementation experiences

To assess implementation experiences, the questionnaire incorporated the acceptability, appropriateness, feasibility, and sustainability constructs from the implementation framework of Proctor and colleagues (25, 39). Acceptability was defined as caregivers' perceptions of whether the materials were agreeable and enjoyable to use, assessed through the statements: “The method was fun to use,” “The method was easy to use,” and “Using the method took little time.” Appropriateness referred to the perceived fit, suitability, and relevance of the materials in encouraging FV intake, measured by ”Using the method fitted well in our daily routine” and “I think the different components of the method matched well together.” Feasibility reflected the extent to which caregivers perceived they could successfully implement the method at home, assessed through “I felt motivated using the method” and “My child was enthusiastic about the method and actively participated in using it.” Sustainability examined the likelihood of continued use within the home setting, evaluated through “I plan to use the method more often in the coming year” and “I will stop using the method after this study.” Other constructs from the framework (i.e., adoption, costs, fidelity, and penetration), were not included, as they were considered less relevant for the study objectives. All implementation aspects were assessed using five-point Likert scales, ranging from 1 (“totally disagree” or “never”) to 5 (“totally agree” or “always”).

##### 2.3.1.2 Caregiver-perceived effects on FV acceptance

The post-test questionnaire included items assessing caregivers' perceived effects of the method on their child's FV acceptance. Specifically, caregivers were asked to evaluate perceived changes in the amount and variety of FVs consumed, as well as the child's enjoyment of eating FVs through the following statements: “After using the method, my child enjoys eating fruit/vegetables more,” “After using the method my child eats more fruit/vegetables,” and “After using the method, my child eats a greater variety of fruit/vegetables.” These aspects were assessed using statements rated on a five-point Likert scale, ranging from 1 (“totally disagree”) to 5 (“totally agree”).

##### 2.3.1.3 Usage of the materials

The questionnaire also included items assessing actual usage factors of the materials to capture how parents implemented the method in practice. Questions addressed dose (i.e., frequency of use), time frame within which the method was applied (if used multiple times), and the eating occasion during which it was implemented. Additionally, caregivers were asked to indicate the number of children in their household who participated, their children's expected satiety level, and whether FVs offered were familiar or unfamiliar to their child. These aspects were assessed through multiple choice questions and some open questions.

##### 2.3.1.4 Socio-demographic characteristics of families and children

Socio-demographic information of both caregivers and children was collected. For caregivers, data were gathered on geographical location (province), country of birth, educational level, and the presence of a second caregiver in the household, assessed through multiple-choice questions. For children, information was collected on age, gender, grade (education), introduction of solid foods, and allergies to FVs. Additionally, food fussiness levels were assessed using 6 items of the Childhood Eating Behavior Questionnaire [CEBQ (40)], scored on a five-point Likert scale items ranging from 1 (“never”) to 5 (“always”).

### 2.4 Study population

The study included self-selected caregivers aged ≥18 years who accessed the materials, had printer access, and used the method at least once prior to completing the questionnaire. No formal age criteria were applied for children; caregivers decided suitability, although the primary target audience of Miffy is typically children aged 0–6 years.

Recruitment was supported by a national media campaign coordinated by WUR. A press release from WUR led to coverage by multiple media outlets, including a web article and interview on RTL Nieuws (www.rtlnieuws.nl), one of the most visited Dutch news platforms, ranked #45 nationally in overall web traffic with approximately 21.7 million monthly visits (41). It also led to a regional radio and web interview on Omroep Gelderland (www.gld.nl; ~3.7 million monthly visits) (42). The release was also picked up by at least 11 additional Dutch websites, including Kek Mama (www.kekmama.nl; ~4 million monthly visits) (43), Zwangerenportaal (www.zwangerenportaal.nl; ~436 thousand monthly visits) (44), Mamaplaats (www.mamaplaats.nl; ~145 thousand monthly visits) (45), and Famme (www.famme.nl; ~137 thousand monthly visits) (46), several of which further shared the content on social media. The campaign was additionally promoted on WUR's and Mercis bv (rights holder Miffy) social media. Posts on Mercis' Instagram received ~1,000 likes each; tweets on X reached 6–10 thousand views with 11–40 reposts; WURs posts on X generated ~1.5 thousand views.

### 2.5 Data analysis

Quantitative analyses were conducted using RStudio (version 4.3.1) (47). Descriptive statistics were used to summarize caregiver and child characteristics, implementation context, and perceived effects on FV consumption behaviors. Categorical variables were presented as frequencies and percentages, and as means and standard deviations (SD) for continuous variables (including Likert-scale items). To enhance interpretability, proportions of agreement and disagreement (scores 4–5 and 1–2, respectively) were calculated for Likert-scale outcomes.

To explore differences by food fussiness levels, mean food fussiness scores were calculated by averaging responses to the six CEBQ food fussiness items, with negatively phrased items reverse-coded. These scores were calculated and analyzed only for questionnaires completed for one child (*N* = 174), as it was not possible to link individual responses to specific children in cases where caregivers reported on multiple children. Based on cut-offs from Steinsbekk et al., children were categorized as having normal (score < 3.0), moderate (score 3.0–3.33), or severe (score >3.33) food fussiness (48). One-way ANOVA was used to test for differences in implementation and perceived effectiveness outcomes across food fussiness groups, with *p* ≤ 0.05 considered statistically significant.

Qualitative data from open-ended responses were analyzed thematically using an inductive approach in QDA Miner (49, 50). A single coder conducted the analysis, which was considered appropriate given that survey responses are generally short, descriptive, and of limited complexity (51). The coder first familiarized themselves with the data, performed open coding, and grouped similar codes into overarching categories and themes, following general principles of thematic analysis as described by Braun and Clarke (52). The resulting themes capture additional contextual information, barriers and facilitators of implementation, and perceived effects.

### 2.6 Ethical approval

This study was approved by the Social Sciences Ethics Committee of WUR (approval number: 2023-081) and conducted in accordance with ethical guidelines. All participants provided (online) informed consent before participation in the study.

## 3 Results

### 3.1 Sample characteristics

A total of 215 caregivers completed the survey. After excluding three respondents who did not provide informed consent and three duplicate entries, data from 209 caregivers were included, yielding a response rate of 0.5% based on 42,913 distributed sticker sheets. Most respondents resided in the western provinces (34%), were born in the Netherlands (96%), were highly educated (67%) and lived with a second caregiver in their household (90%). Caregiver characteristics are summarized in Table 1.

**Table 1:** Demographic characteristics of the survey respondent (*N* = 209).

**Characteristic**	**Value**
**Geographical area of residence** ^*^	
West, *N* (%)	70 (34)
South, *N* (%)	52 (25)
East, *N* (%)	50 (24)
Center, *N* (%)	24 (11)
North, *N* (%)	13 (6)
**Born in the Netherlands**	
Yes, *N* (%)	201 (96)
No, *N* (%)	7 (3)
Prefer not to say, *N* (%)	1 (1)
**Highest educational level** ^**^	
Low, *N* (%)	13 (6)
Middle, *N* (%)	49 (23)
High, *N* (%)	145 (67)
Not applicable or no education, *N* (%)	2 (1)
**Second caregiver in respondent's household**	
Yes, lives with us, *N* (%)	188 (90)
No, lives elsewhere, *N* (%)	7 (3)
No, not present/available, *N* (%)	16 (6)
Prefer not to say, *N* (%)	1 (1)
**Highest educational level of second caregiver** ^***^	
Low, *N* (%)	13 (7)
Middle, *N* (%)	55 (28)
High, *N* (%)	125 (64)
No education or don't know, *N* (%)	5 (3)

^*^Provinces of the Netherlands were grouped into five geographical regions: North (Groningen, Friesland, Drenthe), East (Overijssel, Gelderland), South (Limburg, Noord-Brabant, Zeeland), West (Noord-Holland, Zuid-Holland, and Center (Utrecht, Flevoland).

^**^Education grouped as: low (primary or lower secondary vocational education), middle (general secondary or intermediate vocational education), high (higher professional or academic education at a university or university of applied sciences).

^***^Based on N = 195 caregivers who reported a second caregiver living in the household or elsewhere; education levels grouped as described in footnote ^*^.

Caregivers completed the survey for a total of 246 children. Most filled it out for one child (83%), while 33 (16%) completed it for two children and 2 (1%) for three children. The mean age was 3.3 years (SD = 1.7, range 0–9) and 62% were girls. Most children (66%) were not yet enrolled in school. FV allergies were reported in 6% of cases and 75% had been introduced to solid foods between 4–6 months of age. Food fussiness scores were available for children whose caregiver completed the questionnaire for one child only (*N* = 174). Within this group, the mean food fussiness score was 3.1 (SD = 0.8); 45% showed normal, 42% severe, and 13% moderate food fussiness. An overview of the child characteristics is presented in Table 2.

**Table 2:** Child demographic and food-related characteristics (*N* = 246).

**Characteristic**	**Value**
Age (years), mean (SD)	3.3 (1.7)
**Gender**	
Girls, *N* (%)	152 (62)
Boys, *N* (%)	90 (37)
Prefer not to say, *N* (%)	4 (1)
**School grade**	
Does not go to school yet, *N* (%)	162 (66)
Group 1^*^, *N* (%)	43 (18)
Group 2^*^, *N* (%)	23 (9)
Grade 1 or 2, *N* (%)	18 (7)
**FV allergies**, ***N*** **(%)**	
Yes, *N* (%)	14 (6)
No, *N* (%)	232 (94)
**Started with offering solid foods**	
< 4 months old, *N* (%)	18 (7)
4–6 months old, *N* (%)	185 (75)
>6 months old, *N* (%)	43 (18)
Food fussiness score (CEBQ)^*^, mean (SD)	3.1 (0.8)
Moderate, *N* (%)	23 (13)
Normal, *N* (%)	78 (45)
Severe, *N* (%)	73 (42)

^*^Group 1 and group 2 refer to the Dutch school system and typically correspond to kindergarten years internationally (ages 4–6).

^**^Based on a subsample of N = 174 caregivers who completed the questionnaire for one child only.

### 3.2 Implementation context

Caregivers reported using the method with a mean of 1.5 children simultaneously (SD = 1.42, range 1–17), applying it 6.4 times on average (SD = 6.1, range 1–36) before completing the questionnaire. Among those who used the method more than once, most did so within a few days (72%), primarily during dinner (75%) or as a snack (50%). Most caregivers offered both fruits and vegetables (74%), while fewer provided only vegetables (18%) or only fruits (8%). Foods used were predominantly familiar to the children, with 61 and 58% reporting offering familiar fruits and vegetables, respectively.

Red (91%) and green (89%) were the most offered colors; blue was least used (58%). On average, caregivers included 3.9 out of 5 colors while using the method (SD = 1.3); 45% included all five colors, while only 6% offered just one. The top three items per color were: tomato (54%), bell pepper (53%), and strawberry (35%) for red; carrot (61%), mandarin (28%), and bell pepper (20%) for orange; banana (43%), bell pepper (30%), and corn (14%) for yellow; cucumber (37%), broccoli (30%), and spinach (22%) for green; and blueberry (39%), grape (23%), and eggplant (11%) for blue. Further implementation details are provided in Supplementary File 2.

### 3.3 Acceptability, appropriateness, feasibility, and sustainability of method

Table 3 shows that caregivers evaluated the method very positively, with all implementation outcomes scoring above a mean of 4.0. In terms of acceptability, 97% found it fun, 96% easy to use, and 91% not time-consuming, with only 1%−2% disagreeing across items, regardless of the child's level of food fussiness. More than half selected the highest possible score for fun (57%) and ease of use (51%), while 43% did so for time-efficiency. Appropriateness was also positively evaluated, with 85% indicating that the method fit well into their daily routing and 87% reporting that its components matched well together, with only 2 and 4% disagreeing. Feasibility scores were similarly high: 89% felt motivated to use the method and 85% perceived their child to be enthusiastic, while disagreement remained low (3 and 4%). Lastly, regarding sustainability, 80% intended to continue using the method, supported by a low intention to stop (11%), though disagreement varied more widely (6 and 30%). Although small differences in mean scores were observed across food fussiness groups [e.g., caregiver motivation: 4.2 (0.7)−4.5 (0.6); child enthusiasm: 4.2 (0.9)−4.5 (0.6)]; these were not statistically significant [caregiver motivation: *F*(2, 171) = 195, *p* = 0.15; child enthusiasm: *F*(2, 171) = 1.70, *p* = 0.19]. A marginal difference in stop intention was found between food fussiness groups, with slightly lower scores in caregivers of severely food fussy children (1.9 ± 1.1), compared to those with moderately (2.2 ± 1.0) and normal fussy children (2.3 ± 1.0); this difference approached statistical significance, *F*(2, 1717) = 3.05, *p* = 0.05).

**Table 3:** Opinion of caregivers on various implementation concepts of the method on a 5-point Likert scale ranging from 1 = totally disagree to 5 = totally agree for overall sample and per group of food fussiness.

	**Overall sample (*****N*** = **246)**			**Per group of food fussiness**			**ANOVA *p*-value**
	**Mean (SD)**	**Agree**, ***N*** **(%)**	**Disagree**, ***N*** **(%)**	**Normal, (*****N*** = **78)**	**Moderate, (*****N*** = **23)**	**Severe, (*****N*** = **73)**	
**Acceptability**							
The method was fun to use	4.5 (0.6)	240 (97)	3 (1)	4.6 (0.5)	4.6 (0.5)	4.5 (0.7)	0.67
The method was easy to use	4.4 (0.7)	235 (96)	5 (2)	4.4 (0.7)	4.6 (0.5)	4.5 (0.7)	0.24
Using the method took little time	4.3 (0.7)	225 (91)	4 (2)	4.4 (0.6)	4.4 (0.6)	4.5 (0.8)	0.40
**Appropriateness**							
Using the method fitted well into our daily routine	4.2 (0.8)	209 (85)	11 (4)	4.1 (0.8)	4.2 (0.9)	4.3 (0.8)	0.30
I think the different components of the method matched well together	4.2 (0.8)	213 (87)	6 (4)	4.2 (0.8)	4.3 (0.6)	4.3 (0.7)	0.51
**Feasibility**							
I felt motivated using the method	4.3 (0.8)	218 (89)	7 (3)	4.2 (0.7)	4.3 (0.7)	4.5 (0.6)	0.15
My child was enthusiastic about the method and actively participated in using it	4.2 (0.9)	209 (85)	11 (4)	4.3 (0.7)	4.5 (0.6)	4.2 (0.9)	0.19
**Sustainability**							
I plan to use the method more often in the coming year	4.1 (0.9)	198 (80)	15 (6)	4.0 (0.8)	4.0 (1.2)	4.3 (1.0)	0.24
I will stop using the method after this study	2.2 (1.0)	26 (11)	171 (70)	2.3 (1.0)	2.2 (1.0)	1.9 (1.1)	0.05

### 3.4 Perceived effects by caregiver's post-use

As shown in Table 4, more than half of caregivers reported increased child enjoyment of fruits (53%) and vegetables (59%) after using the method, with 9 and 14% selecting the highest possible rating and only a small proportion disagreeing (12 and 15%, respectively). Approximately 40% perceived a positive effect on the amount of fruit (40%) and vegetable (53%) consumption, and 46 and 53% on fruit and vegetable variety, while fewer than one in five disagreed (19%−23% across items). On average, caregivers were slightly positive about the method's effectiveness, with mean scores just above 3 on a 5-point scale. Perceived effects were consistently higher for vegetables than fruits across all items based on visual inspection and this pattern was observed across all levels of food fussiness. No significant differences were observed in perceived effectiveness between children classified as normal, moderate, or severe fussy eaters.

**Table 4:** Caregiver-experienced changes in the child's eating behavior after using the method on a 5-point Likert scale ranging from 1 = totally disagree to 5 = totally agree for overall sample and per group of food fussiness.

**Fruit (*N* = 201)**	**Overall sample**			**Per group of food fussiness**			**ANOVA *p*-value**
	**Mean (SD)**	**Agree**, ***N*** **(%)**	**Disagree**, ***N*** **(%)**	Normal^*^, (***N*** = **78)**	Moderate^**^, (***N*** = **23)**	Severe^***^, (***N*** = **73)**	
After using the method, my child enjoys eating fruit more	3.5 (0.9)	107 (53)	24 (12)	3.5 (1.0)	3.5 (0.6)	3.4 (0.9)	0.35
After using the method, my child eats more fruit	3.2 (1.0)	81 (40)	46 (23)	3.2 (1.0)	3.5 (0.9)	3.1 (1.0)	0.86
After using the method, my child eats a greater variety of fruits	3.3 (1.0)	92 (46)	38 (19)	3.4 (0.9)	3.5 (0.71)	3.3 (1.0)	0.57
**Vegetables (*****N*** = **226)**							
After using the method, my child enjoys eating vegetables more	3.6 (1.0)	133 (59)	33 (15)	3.7 (0.9)	3.6 (1.0)	3.6 (1.0)	0.37
After using the method, my child eats more vegetables	3.4 (1.0)	108 (48)	42 (19)	3.4 (1.0)	3.7 (0.8)	3.4 (1.0)	0.73
After using the method, my child eats a greater variety of vegetables	3.5 (1.0)	120 (53)	42 (19)	3.5 (0.9)	3.8 (0.7)	3.5 (1.0)	0.37

^*^Normal food fussiness group: fruit items (N = 67), vegetables items (N = 70); based on participants who offered the respective foods while using the method.

^**^Moderate food fussiness group: fruit items (N = 18), vegetable items (N = 21); based on participants who offered the respective foods while using the method.

^***^Severe food fussiness group: fruit items (N = 58), vegetable items (N = 67); based on participants who offered the respective foods while using the method.

### 3.5 Complementary qualitative insights on implementation and impact

Caregivers' open-ended comments (*N* = 135) provided additional qualitative insights into implementation and effectiveness experiences, relating to four key themes: *perceived effects* (38/135, 28%), *implementation experiences* (30/135, 22%), *barriers and facilitators to use* (39/135, 29%), and *recommendations for improvement* (26/135, 19%). Additional perceived effects not explicitly captured in the questionnaire included increased willingness and motivation to try new FVs (66%), as one noted: “*Although my daughter is not yet eating more vegetables, the method definitely helps lower the threshold for trying something for the first time!”*. Additional perceived effects included a more positive mealtime atmosphere (13%) and broader learning outcomes unrelated to eating behavior, such as recognition of different colors and FV types (13%). Comments on implementation experiences highlighted variations in how the method was used (40%), including combining it with other FV promotion strategies or incentives (e.g., a gift when completing the poster), while others engaged children in grocery shopping (27%): “*Tomorrow, we're taking the rainbow to the supermarket, and we will look for fruits and vegetables for all the colors together!”*. Reported barriers to use included concerns about sustaining motivation over time once novelty is diminished or the sticker reward was no longer provided (38%): “*I am afraid the effect will fade, the fun eventually wears off* .” Some caregivers noted that the method was less suitable for children perceived as too young or too old (18%): “*My son is 2.5 years old. I think that's too young to work with this method. He enjoyed it, but didn't really understand it yet. In about six months, I'd like to try it again.”* Facilitating factors included the appeal of the familiar Miffy character (23%): “*It helped that the method used a well-known figure, this immediately boosted the motivation for our little one*.” Several caregivers also emphasized that more time and repeated practice might be necessary to observe behavioral changes (5%): “*I think it just takes more time and practice before your child actually starts eating the new foods*.” Finally, recommendations for improvement of the method focused on suggestions for material adaptations (62%), such as introducing additional sticker colors or offering alternative designs featuring other popular children's (cartoon) characters.

## 4 Discussion

This study examined caregivers' experiences with the in-home use of “Miffy eats the rainbow!” and their perceptions of its effectiveness, following free nationwide sticker distribution in the Netherlands. Despite broad dissemination, the response was limited, with 209 caregivers completing the questionnaire. These self-selected caregivers demonstrated flexible implementation, most often during dinner or snack times, using familiar red and green FVs. Implementation experiences were rated highly positive across all domains, regardless of children's food fussiness levels. Perceived effects on children's enjoyment, amount, and variety of FV consumption were slightly positive, with somewhat higher ratings for vegetables. Qualitative responses provided complementary insights, highlighting additional benefits, diverse implementation strategies, barriers, facilitators, and suggestions for refinement.

### 4.1 Implementation potential

Self-selected caregivers rated the method as highly acceptable, appropriate, feasible, and sustainable for in-home use, with over 90% agreeing it was fun, easy to use, and time efficient. These factors are well-established facilitators of sustained implementation (53), particularly in home settings where time constraints and competing demands often hinder FV promotion (54–58). Perceived ease of use, in particular, is known to enhance caregiver uptake and continued use (54, 59). Interestingly, implementation ratings remained consistently high across all levels of food fussiness, it being a well-known barrier for caregivers to improve FV intake (59–61). This may indicate that the method's simplicity and playful design helped overcome initial resistance even in more selective eaters, supported by caregiver comments referencing the appeal of the familiar cartoon character and playful approach. These findings align with previous research showing that recognizable characters and gamified elements can enhance both caregiver and child engagement and promote FV selection and intake (62–64) and suggest that the method may offer a valuable tool for caregivers seeking positive ways to support selective eaters.

Although not significant, caregivers of severely fussy children rated feasibility and continued use particularly high. This may reflect a greater caregiver need for practical and sustainable strategies to improve their child's FV intake, despite lower enthusiasm from their child; a hypothesis that warrants further investigation. Caregivers of moderately fussy children also rated these items positively, but their children appeared more enthusiastic than those in the normal and severely fussy groups. This suggests that moderately fussy children, who may still require support in developing FV acceptance yet appear more receptive, could be a particularly promising target group for a playful, low-threshold intervention to improve FV intake. However, given the small size of this subgroup in our sample, further research is needed to substantiate these findings.

In addition to the high implementation scores, caregivers reported slightly positive perceived effects on children FVs consumption pleasure, amount, and variety, with marginally stronger results for vegetables than fruits; likely reflecting children's greater initial liking of fruits (65, 66). Overall modest effects may be due to the short duration of use and the fact that many caregivers offered mostly familiar foods, leaving limited room for improvement. While the method draws on well-established strategies, such as repeated exposure and modeling, these are most effective with unfamiliar foods and require sustained, consistent application (12, 13). Several caregivers also noted that more time and practice would be needed to achieve meaningful changes in attitude and behavior, which aligns with evidence suggesting that FV acceptance may require 5 (67) to 27 (68) taste exposures and that insufficient exposure may have limited the observed effects. The impact of modeling is also known to increase with frequent and consistent exposure to role models, whether through the caregiver or a familiar character such as Miffy (17, 62, 69). In this study, caregivers had full autonomy in how and when to apply the method and complete the questionnaire to maintain a low-threshold, real-world approach, which allowed for a low-threshold, real-world application, but may have diluted the observed effects. In addition, because the questionnaire focused on experiences rather than on assessing mechanisms of action, the use of repeated exposure, rewards, modeling (through Miffy and/or caregivers) was not examined in detail. Future studies could more closely investigate how and how often these behavioral strategies are applied to better under understand their contribution to implementation and effectiveness.

Notably, while the questionnaire assessed pleasure, variety, and amount of FV consumption, willingness to try new foods emerged as a perceived additional effect in qualitative responses. This suggest that the method may have supported early-stage willingness to try at home, an effect potentially not fully captured quantitatively and warranting further investigation (16). The sticker-based reward component, while not isolated in this study, may have contributed to this initial excitement and engagement to taste. However, some caregivers noted that its motivational effect might diminish over time, reflecting broader concerns about reliance on external rewards than fostering intrinsic motivation (65, 66). Although previous research has demonstrated long-term positive effects of reward-based strategies on FV behaviors (17, 65), their specific role in sparking initial willingness to try remains underexplored and requires further study (19).

Despite the encouraging evaluations suggesting good in-home implementation potential and modest perceived effects, these results should be interpreted with some caution. Most questionnaires were completed within only a few days of starting the method, meaning that reported experiences largely reflect first impressions. While initial responses are often positive, sustaining engagement over time is typically more challenging in nutrition interventions (59, 70, 71). Future research should therefore examine longer-term use and adherence to better understand the durability of these positive experiences.

### 4.2 Value and limitation of citizen science approach

A notable and unexpected finding of this study was the extremely low response rate, despite free nationwide distribution of stickers through widely used retail chains in the Netherlands and extensive promotion of the citizen science initiative. Additional measures were implemented to enhance accessibility, including email requests, direct questionnaire access, and an incentive upon completion. Despite these efforts, participation remained very limited, suggesting that factors such as limited perceived relevance, lack of personal connection to the research aim, or competing demands on caregivers' time may have outweighed the motivation to engage (31). This highlights a key challenge in citizen science: even with broad dissemination and incentives, voluntary participation may be difficult to achieve without stronger personal or community involvement.

This challenge mirrors broader patterns observed in citizen science projects, particularly in contributory projects where participants are primarily involved in data collection. A scoping review by Marks and colleagues (31) found that 61% (21/33) of such prevention-focused initiatives reported sample sizes below 100, underscoring the persistent difficulty of mobilizing large-scale participation even in accessible projects. This reflects a broader trade-off: while contributory projects aim for broad reach, limited perceived engagement may hinder recruitment and retention. In contrast, smaller collaborative projects engage participants across multiple research stages, including design, analysis, and dissemination, enhancing commitment, but constraining scalability (72).

Importantly, the low response rate in the present study raises concerns about nonresponse bias, where non-respondents may differ systematically from participants (73). Caregivers who were particularly motivated, either because of highly positive or highly negative experiences, may have been more likely to respond, potentially skewing the results and obscuring the range of experiences. Several practical barriers may also have reduced participation. For example, the need to download and print the poster could have excluded families without easy access to printing facilities. In addition, although the sticker sheets were made widely available through national retail chains, this distribution channel may not sufficiently reach less engaged or socioeconomically disadvantaged groups. Such factors may contribute to the underrepresentation of harder-to reach families (74), including those who already face barriers to adequate FV consumption (70). Providing printed materials directly and using more inclusive dissemination channels, including child health clinics, community centers, or schools, may help ensure more equitable participation in future initiatives.

As this study employed a contributory citizen science approach, in which participants involvement was limited to providing data, opportunities for more substantive engagement were inherently restricted. In the context of promoting FV intake in young children, higher-intensity citizen science approaches, such as involving caregivers in co-developing materials, refining instructions, or contributing to the interpretation of findings, could have increased the perceived relevance and accessibility of the method and may have supported broader and more diverse participation. Such approaches, however, require more sustained interaction and typically reduce scalability. Future research may therefore consider incorporating targeted collaborative elements, such as structured feedback opportunities or participant reflections on interim results, while maintaining a design that supports broad dissemination. Additionally, innovative tools, including mobile apps, digital platforms, or gamified approaches successfully used in other scientific fields (75–80), may offer promising alternatives to traditional online questionnaires, helping to strengthen collaborative aspects in larger-scale contributory public health citizen science initiatives.

### 4.3 Strengths and limitations

A key strength of this study is its unique design, involving free, nationwide distribution of the intervention materials, reflected in responses received from caregivers across all provinces of the Netherlands. To our knowledge, this is the first study of its kind, with over 43,000 sticker sets disseminated, which offered substantial potential for large-scale data collection. Another strength is that caregivers were given full autonomy in how they integrated the method into their daily routine. Informed by self-determination theory (81), this flexibility was expected to support intrinsic motivation and enhance ecological validity by closely reflecting real-world use. Additionally, the qualitative analysis of open-ended responses alongside close-ended questionnaire items allowed for deeper contextual understanding and complementary qualitative insights.

However, several limitations should be noted. While the flexible design enhanced ecological validity, it also introduced variability in how and to what extent the method was applied, complicating interpretation of the observed effects. Moreover, data were collected mostly shortly after implementation, limiting conclusions about sustained use and long-term implementation and effectiveness experiences. Additionally, our sample's predominance of highly educated, non-immigrant, two-caregiver households suggest potential selection bias, thereby limiting generalizability to more diverse populations. The reliance on caregiver-reported data, without objective tracking of method use, further restricts interpretation of implementation fidelity and intensity. Furthermore, in households where the method was used with multiple children, but the questionnaire was completed for only one, caregivers' responses may have reflected experiences with all participating children. As the questionnaire referred to one identified child, any such influence is expected to be minimal. Additionally, it was not possible to determine exactly how many sticker sheets and posters were distributed or used, limiting our ability to estimate true reach. Finally, the one-group post-test only design prevents drawing conclusions regarding effectiveness.

## 5 Conclusion

This study demonstrates that the “Miffy eats the rainbow!” method was perceived by self-selected caregivers as highly acceptable, appropriate, feasible, and sustainable for in-home use, with consistently positive rating across different levels of child food fussiness. These findings highlight the potential of a simple, colorful method incorporating repeated exposure, modeling, and non-food rewards strategies to support FV promotion in home settings. However, the study also revealed a very low response rate, despite nationwide distribution and extensive promotion, underscoring the challenge of mobilizing large-scale participation in contributory citizen science. The self-selected nature of respondents further raises the risk of selection and non-response bias, likely overrepresenting motivated or positively inclined caregivers. Future research should explore strategies to enhance inclusive engagement in citizen science initiatives and assess the methods' longer-term implementation and effectiveness across more diverse and representative populations.

## Data Availability

The raw data supporting the conclusions of this article will be made available by the authors, without undue reservation.
