# Supplementary material for: Caregivers' perspectives on the in-home implementation and effectiveness of “Miffy eats the rainbow!”: a colorful, modeling- and reward-based intervention to improve fruit and vegetable intake in children
Source: Front Public Health. 2026 Jan 12;13:1663525. doi: 10.3389/fpubh.2025.1663525 (PMC12891986; doi:10.3389/fpubh.2025.1663525)
Supplement: Supplementary file 1 [file Table_1.docx]

Supplementary Material

# Supplementary File 1: Post-test questionnaire

*Items marked with an asterisk (*) were mandatory and had to be completed before participants could submit the questionnaire.*

*Items marked with two asterisks (**) were conditionally displayed and appeared only when relevant based on participants’ previous responses.*

We would like to investigate whether the 'Miffy eats the rainbow!' method helps your child to eat more (varieties of) fruits and vegetables. Have you completed the 'Miffy eats the rainbow!' method (reading the story, tasting fruits and vegetables, and then sticking stickers) at least once? If so, you can start the questionnaire.

**1. General information**

| In which province do you live?* | - Drenthe - Flevoland - Friesland - Gelderland - Groningen - Limburg - Noord-Holland - Noord-Brabant - Overijssel - Utrecht - Zuid-Holland - Zeeland |
| --- | --- |
| What is your highest completed level of education? * | - Primary school - VMBO, VBO, MAVO, MBO level 1 - HAVO, VWO, MBO level 2-4 - University of applied sciences or university’s bachelor degree - University master’s degree - Not applicable |
| Were you born in the Netherlands? * | - Yes - No, namely: _________ - Prefer not to say |
| Does a second parent or caregiver live with your child at home, in addition to yourself? | - Yes, lives with us at home - No, lives elsewhere - No, there is no other parent or caregiver involved - Prefer not to say |
| What is the highest completed level of education of the second parent or caregiver of your child? ** | - Not applicable - No education - Primary school - VMBO, VBO, MAVO, mbo-level 1 - HAVO, VWO, mbo-level 2-4 - University of applied sciences or university’s bachelor degree - University’s master degree - I don’t know |
| Was the second parent or caregiver born in the Netherlands? ** | - Not applicable - Yes - No, namely: _________ - I don’t know - Prefer not to say |

If multiple children participated with you in the 'Miffy eats the rainbow!' method, you can choose to answer the questions for one child or for each child separately.

| With how many children did you test the 'Miffy Eats the Rainbow' method? * | _____ children |
| --- | --- |
| For how many children would you like to complete the questionnaire? If you want to complete it for more than one child, the following questions will be repeated for each child. ** | _____ children |
| What is your child’s age? * | _____ years |
| What is your child’s gender? | - Boy - Girl - Prefer not to say |
| What school year/class is your child in? * | - My child does not attend school yet - Group 1 (Dutch system) - Group 2 (Dutch system) - Group 3 (Dutch system) - Group 4 (Dutch system) |
| Does your child have any allergies or intolerances to fruit and/or vegetable types? * | □ Yes, namely: ____  □ No  □ Prefer not to say |
| When did you start offering solid foods to your child? | - My child was younger than 4 months. - My child was between 4–6 months old. - My child was older than 6 months. |

To what extent is your child willing to try new foods? The following statements are about your experiences.

|  | **Never** | **Rarely** | **Sometimes** | **Often** | **Always** |
| --- | --- | --- | --- | --- | --- |
| My child is interested in tasting food he/she has never tried before * | □ | □ | □ | □ | □ |
| My child enjoys eating many different types of food * | □ | □ | □ | □ | □ |
| My child initially refuses unfamiliar food * | □ | □ | □ | □ | □ |
| It is difficult to please my child with food * | □ | □ | □ | □ | □ |
| My child decides he/she dislikes food even without tasting it * | □ | □ | □ | □ | □ |
| My child likes to try new food * | □ | □ | □ | □ | □ |

1. **Use of the ‘Miffy eats the rainbow!’ method**

This second part of the questionnaire is about how you have used the method.

| How many times have you used the ‘Miffy eats the rainbow!’ method so far? * | _____ times |
| --- | --- |
| How much time passed between the first and last time you used the ‘Miffy eats the rainbow!’ method? ** | - Not applicable - One to a few days - One week - Two or three weeks - More than a month |
| At what time of the day did you use the ‘Miffy eats the rainbow!’ method?  Multiple options possible | - Breakfast - Lunch - Lunch - Snack - Random |
| Did your child eat just before using the ‘Miffy eats the rainbow!’ method? *,** | - No - Yes, a snack within the past hour - Yes, a snack more than an hour earlier - Yes, a meal within the past hour - Yes, a meal more than an hour earlier |
| Did you – besides offering the fruits and vegetables using the ‘Miffy eats the rainbow!’ method – change anything else in what your child was given as a snack or meal on the same day? *,** | □ Yes, offered a different snack/meal  □ Yes, skipped a snack/meal  □ No |

We would also like to know which types of fruits and vegetables you offered your child.

| What did you offer your child when using the method? * | - Vegetables - Fruit - Both |
| --- | --- |
| Which colors of fruit and/or vegetables did you offer? You may select multiple options. * | - Red - Orange - Yellow - Green - Blue |
| For the color RED, I offered the following fruits and/or vegetables: * | _____________________________________________ |
| For the color ORANGE, I offered the following fruits and/or vegetables: * | _____________________________________________  ___________________________________________ |
| For the color YELLOW, I offered the following fruits and/or vegetables: * | _____________________________________________  ___________________________________________ |
| For the color BLUE, I offered the following fruits and/or vegetables: * | _____________________________________________  ___________________________________________ |
| For the color GREEN, I offered the following fruits and/or vegetables: * | _____________________________________________  ___________________________________________ |
| Offered fruits: [responses]  My child had eaten these fruit types before *,** | - Yes - No, he/she had never eaten these fruit(s) before: _____________________________________ |
| Offered vegetables: [responses]  My child had eaten these vegetable types before *,** | - Yes - No, he/she had never eaten these fruit(s) before: _____________________________________ |

The following statements are about your experience using the 'Miffy eats the rainbow!' method. Please indicate for each statement to what extent you agree or disagree.

|  | **Strongly disagree** | **Disagree** | **Neutral** | **Agree** | **Strongly agree** |
| --- | --- | --- | --- | --- | --- |
| The method was fun to use * | □ | □ | □ | □ | □ |
| The method was easy to use * | □ | □ | □ | □ | □ |
| Using the method takes little time * | □ | □ | □ | □ | □ |
| The method fits well into our (daily) routine * | □ | □ | □ | □ | □ |
| I think the different components of the method fit well together * | □ | □ | □ | □ | □ |
| I felt motivated to use the method * | □ | □ | □ | □ | □ |
| My child was enthusiastic about the method and actively participated in using it * | □ | □ | □ | □ | □ |
| I will stop using the method after this study * | □ | □ | □ | □ | □ |
| I plan to use the method more often in the coming year * | □ | □ | □ | □ | □ |

1. **Effects of using ‘Miffy eats the rainbow!’ method**

This final section is about how well the 'Miffy eats the rainbow!' method helped you encourage your child to eat fruits and vegetables. First, we ask about the effect of the method on your child’s fruit consumption.

|  | **Strongly disagree** | **Disagree** | **Neutral** | **Agree** | **Strongly agree** |
| --- | --- | --- | --- | --- | --- |
| My child eats **more** fruit after the method *, ** | □ | □ | □ | □ | □ |
| My child **enjoys** eating fruit more after the method *, ** | □ | □ | □ | □ | □ |
| My child eats a greater **variety** of fruit after the method *, ** | □ | □ | □ | □ | □ |

Now we ask about the effect of the ‘Miffy eats the rainbow!’ method on your child’s vegetable consumption.

|  | **Strongly disagree** | **Disagree** | **Neutral** | **Agree** | **Strongly agree** |
| --- | --- | --- | --- | --- | --- |
| My child eats **more** vegetables after the method *, ** | □ | □ | □ | □ | □ |
| My child **enjoys** eating vegetables more after the method *, ** | □ | □ | □ | □ | □ |
| My child eats a greater **variety** of vegetables after the method *, ** | □ | □ | □ | □ | □ |

1. Other

Do you have any other comments about your experience using the ‘Miffy eats the rainbow!’ method?
