# Supplementary material for: Caregivers' perspectives on the in-home implementation and effectiveness of “Miffy eats the rainbow!”: a colorful, modeling- and reward-based intervention to improve fruit and vegetable intake in children
Source: Front Public Health. 2026 Jan 12;13:1663525. doi: 10.3389/fpubh.2025.1663525 (PMC12891986; doi:10.3389/fpubh.2025.1663525)
Supplement: Supplementary file 2 [file Table_2.docx]

Supplementary Material

# Supplementary File 2: Details on implementation context

Table 1. Details on implementation context and characteristics (N = 246).

| **Characteristic** | | **Value** |
| --- | --- | --- |
| **Frequency of use ^a^, mean (SD)** | | 6.4 (6.1) |
| **Duration ^a^, N (%)** | |  |
|  | One to a few days | 151 (61) |
|  | A week | 24 (10) |
|  | Two or three weeks | 28 (11) |
|  | More than a month | 7 (3) |
|  | Not applicable | 36 (15) |
| **Meal context ^a^, mean (SD)** | | 1.9 (1.1) |
|  | Breakfast, N (%) | 35 (14) |
|  | Lunch, N (%) | 79 (32) |
|  | Dinner, N (%) | 186 (75) |
|  | Snack, N (%) | 122 (50) |
|  | Random, N (%) | 49 (20) |
| **Food intake prior to using method ^a^, N (%)** | |  |
|  | No | 16 (7) |
|  | Yes, a snack within the hour | 6 (2) |
|  | Yes, a snack more than an hour ago | 5 (2) |
|  | Yes, a meal within the hour | 4 (2) |
|  | Yes, a meal more than an hour ago | 5 (2) |
|  | Not answered or not applicable | 210 (85) |
| **Meal adjustments ^a^, N (%)** | |  |
|  | Yes, offered another snack/meal | 4 (2) |
|  | Yes, skipped a snack/meal | 1 (1) |
|  | No | 32 (13) |
|  | Not answered or not applicable | 209 (85) |
| **Types of foods offered ^a^, N (%)** | |  |
|  | Fruits | 20 (8) |
|  | Vegetables | 45 (18) |
|  | Fruits and vegetables | 181 (74) |
| **Colors offered ^a^, mean (SD)** |  | 3.9 (1.2) |
|  | Red, N (%) | 224 (91) |
|  | Orange, N(%) | 202 (82) |
|  | Yellow, N (%) | 180 (73) |
|  | Green, N (%) | 218 (89) |
|  | Blue, N (%) | 143 (58) |
| **Familiarity with offered vegetables ^a^, N (%)** |  |  |
|  | Yes | 104 (60) |
|  | No | 53 (30) |
|  | Not answered or not applicable | 17 (10) |
| **Familiarity with offered fruits ^a^, N (%)** |  |  |
|  | Yes | 105 (60) |
|  | No | 40 (23) |
|  | Not answered or not applicable | 29 (17) |
