# Supplementary material for: Caregivers' perspectives on the in-home implementation and effectiveness of “Miffy eats the rainbow!”: a colorful, modeling- and reward-based intervention to improve fruit and vegetable intake in children
Source: Front Public Health. 2026 Jan 12;13:1663525. doi: 10.3389/fpubh.2025.1663525 (PMC12891986; doi:10.3389/fpubh.2025.1663525)
Supplement: Supplementary file 3 [file Table_3.docx]

Supplementary Material

# Supplementary File 3: Use of Generative AI

During the editing process of this manuscript, the authors used ChatGPT (GPT-4, April 2024 version) developed by OpenAI to improve clarity, conciseness, and scientific tone. The AI was used solely for language editing and formatting purposes. Prompts included requests such as:

- “Make this sentence more concise”;
- “Improve coherency and flow”;
- “Clarify meaning while retaining scientific tone”.
